# Risk factors associated with disease severity among children hospitalised with community-acquired pneumonia in Angola

**DOI:** 10.7189/jogh.16.04138

**Published:** 2026-04-30

**Authors:** Linda Reitala, Maija-Katri Lehto, Satu Kekomäki, Miia Salaspuro, Elizabete dos Anjos, Silvia Silvestre, Elsa Gomes, Manuel Leite Cruzeiro, Asuncion Mejias, Octavio Ramilo, Tuula Pelkonen

**Affiliations:** 1 New Children’s Hospital, Pediatric Research Center, Helsinki University Hospital and University of Helsinki, Helsinki, Finland; 2 Hospital Pediátrico David Bernardino, Luanda, Angola; 3 St. Jude Children’s Research Hospital, Memphis, Tennessee, USA

## Abstract

**Background:**

Community-acquired pneumonia (CAP) is a major cause of paediatric mortality worldwide, disproportionately affecting children in low- and middle-income countries. Yet, to date, no studies have examined the risk factors for severe CAP in Angolan children. We aimed to identify these risk factors to improve CAP-associated morbidity and mortality.

**Methods:**

We conducted a prospective, observational study enrolling a convenience sample of children aged two months to 13 years hospitalised with CAP in the Hospital Pediátrico David Bernardino in Luanda, Angola. We included healthy children and those with asthma, human immunodeficiency virus, or sickle cell disease. We used multivariable logistic regression to assess risk factors associated with mortality as the primary outcome and prolonged hospitalisation as the secondary outcome.

**Results:**

From September 2019 to May 2021, we enrolled 372 patients hospitalised with CAP, confirmed by chest x-ray. Multivariable analysis identified age <1 year (adjusted odds ratio (aOR) = 7.51; 95% confidence interval (CI) = 1.72–34.12), seizures (aOR = 10.62; 95% CI = 1.17–80.71), and C-reactive protein ≥150 mg/L (aOR = 27.77; 95% CI = 4.48–575.26) as risk factors for mortality, while cough prior to hospitalisation was a protective factor (aOR = 0.05; 95% CI = 0.01–0.2). Cooking at home with coal or wood (aOR = 2.05; 95% CI = 1.13–3.80), malnutrition (aOR = 2.15; 95% CI = 1.17–4.01), inability to drink on admission (aOR = 2.43; 95% CI = 1.22–5.00), diminished breath sounds on auscultation (aOR = 2.67; 95% CI = 1.49–4.82), and pleural effusion on chest x-ray (aOR = 3.99; 95% CI = 2.23-7.27) were risk factors for prolonged hospitalisation (>7 days).

**Conclusions:**

We identified various demographic, environmental, clinical, laboratory, and radiologic findings as key risk factors for severe CAP in Angolan children.

Community-acquired pneumonia (CAP) remains a significant cause of death among children worldwide, with disproportionately high mortality in low- and middle-income countries (LMICs) [1]. Implementation of feasible and effective interventions – including vaccinations, simplified treatment guidelines, improved antibiotic access, and measures to enhance hygiene, nutrition, and living conditions – has reduced pneumonia mortality in children younger than five years globally from 13.6 per 1000 live births in 2000 to 3.8 per 1000 live births in 2019 [1,2]. The addition of the 13-valent pneumococcal conjugate vaccine to the national immunisation programme in Angola in 2013 was a promising step towards further reducing paediatric CAP mortality in this population, with the coverage of the final dose estimated at 48% in 2021 [3]. Nonetheless, in Angola, under-five mortality rate attributable to pneumonia was still 13 per 1000 live births in 2018 [4], indicating a continued need for effective interventions.

Although CAP is a major cause of serious illness worldwide, validated disease severity criteria for children are lacking [5–7]. Studies conducted in LMICs have identified host- and disease-related risk factors associated with childhood pneumonia mortality, but fewer studies have explored the role of sociodemographic, epidemiologic, and laboratory factors [8]. Additionally, factors associated with prolonged hospitalisation (*i.e.* length of stay (LOS)) in paediatric CAP have been understudied in LMICs [9], despite being a key determinant of the overall cost of treating severe pneumonia cases in those settings [10].

Despite the high mortality rates associated with childhood pneumonia in Angola, to date, no studies have examined the risk factors for severe CAP in Angolan children. We aim to fill that gap in knowledge by identifying these risk factors to improve clinical outcomes.

## METHODS

### Study design and patients

We conducted a prospective observational study on a convenience sample in the Hospital Pediátrico David Bernardino (HPDB), Angola, from September 2019 to May 2021. The HPDB, Angola’s largest paediatric hospital with 350 beds, has a limited intensive care unit of 20 beds that is often at full capacity.

Children aged two months to 13 years hospitalised with CAP were eligible for inclusion. We included previously healthy children, those with human immunodeficiency virus (HIV) or sickle cell disease, and those with asthma. We excluded patients with significant pre-existing conditions (*e.g.* chronic lung or cardiac disease, neuromuscular disease, immunodeficiency, malignancy, cystic fibrosis) and those receiving immunomodulatory agents (*e.g.* corticosteroids >2 weeks within the previous six weeks), as these factors could independently affect pneumonia severity. We also excluded patients with a primary diagnosis of bronchiolitis or hospitalisation within the previous seven days to focus on CAP. We enrolled patients within 48 hours of admission following written informed consent from their guardians or parents.

Pneumonia was diagnosed according to the Center for Disease Control and Prevention Emergency Partners Information Connection study criteria [11], based on evidence of an acute infection, signs or symptoms of respiratory illness, and radiologic confirmation of pneumonia (Table S1 in the **Online Supplementary Document**). The radiologic diagnosis of pneumonia per attending physician was adequate for inclusion in the study, while all radiologic variables used in the analyses were assessed based on an independent radiological review performed at Helsinki University Hospital. We followed the STROBE guidelines for the reporting of this study (Table S2 in the **Online Supplementary Document**) [12]

### Clinical data and microbiological evaluation

We obtained different laboratory studies per standard of care in children hospitalised with CAP at HPDB during the study period, including blood culture, complete blood cell count, and haemoglobin. Samples of pleural fluid were analysed by routine bacterial culture. We also recorded malaria, tuberculosis, HIV antibody, and sickle cell screening tests, if available. In addition, we obtained capillary blood samples per study protocol to measure C-reactive protein (CRP) at enrollment and on day four of illness. Point-of-care test (QuikRead go wrCRP, Aidian Oy, Espoo, Finland) was used to measure CRP concentrations.

The study nurses filled in a predefined study questionnaire. We used standardised study forms to collect demographic parameters, patients’ medical history, and clinical and radiographic data. Clinical data included administered medications, intravenous fluids, blood transfusions, respiratory support, laboratory results, clinical outcomes, and daily clinical findings (*i.e.* temperature, respiratory rate, and blood oxygen saturation). We reviewed patient healthcare records for additional data.

We defined cough before hospitalisation as any caregiver-reported cough preceding admission. We assessed malnutrition using clinical criteria, in line with the World Health Organization recommendations, as it was available for more patients than z-scores and captures features such as muscle wasting and oedema not reflected in weight alone. We defined altered consciousness as a Glasgow Coma Scale score of <15, and coma as a score of <8. We used the Pediatric Early Warning Score scoring system to adjust for blood pressure, heart rate, and respiratory rate, categorising the scores as: normal (0 points), moderate increase/decrease (1 point), severe increase/decrease (≥2 points). We entered the data into a secure Research Electronic Data Capture system. During the study period, the attending physician decided on the discharge of patients, as there were no standard guidelines at HPDB.

### Statistical analysis

We selected mortality as the primary outcome and prolonged hospitalisation (LOS>7 days) as the secondary outcome, based on a literature review and expert opinion. We defined prolonged hospitalisation as LOS>7 days, as it represented the median (MD) LOS in the cohort and a pragmatic measure of extended inpatient care. We did not conduct a formal sample size or power calculation, since mortality was not the primary endpoint of the original study. We first identified candidate predictors based on previous literature and expert opinion. We then analysed their associations with the outcomes using exploratory analyses. We excluded variables with >25% missing values. We did not perform imputation and assumed missing data to be missing at random. We did not conduct sensitivity analyses to assess alternative missingness assumptions.

We assessed the normality of continuous variables using the Shapiro-Wilk test. As none were normally distributed, we used the Mann-Whitney U test for comparisons. For categorical variables, we used Fisher exact test for small samples and the χ^2^ test for larger samples.

Subsequently, we used univariable and multivariable logistic regression models to further analyse the effects of those statistically significant predictors on the outcomes. As we aimed to identify potential risk factors rather than develop a predictive model, we included predictors with *P* < 0.05 in univariable analyses in multivariable models using forward selection. We built the models in a stepwise manner by adding candidate predictors one at a time to the base model. After each addition, we evaluated model performance using several standard performance metrics. First, we used Nagelkerke’s R^2^ to examine the model goodness-of-fit, which ranges from 0 to 1, with higher values (*e.g.*>0.2) indicating better fit. Second, we evaluated the discriminative ability of the final multivariable regression models using the AUC, where values >0.7 indicate acceptable discrimination. To further evaluate model performance, we calculated overall accuracy and precision. Accuracy describes the overall proportion of correctly classified cases, whereas precision indicates how often a positive prediction corresponds to a true positive outcome. Lastly, we evaluated the overall performance with the Brier score, which quantifies the accuracy of probabilistic predictions, reflecting both discrimination and calibration. The score ranges from 0 to 1, where 0 indicates perfect accuracy and values <0.2 are generally considered acceptable.

We used correlation matrices and variance inflation factor values to assess multicollinearity among predictors. We considered <0.7 correlations and <5 variance inflation factor values acceptable. Correlations between the independent variables ranged from −0.238 to 0.330, and variance inflation factor values ranged from 1.06 to 1.88, indicating no significant multicollinearity.

We used *R*, version 4.2.1 (R Core Team, Vienna, Austria) for all analyses. We considered a *P*-value <0.05 statistically significant. We used artificial intelligence, specifically Copilot, in the writing process to check spelling and grammar, suggest synonyms or rephrasing, and review and verify the *R* code.

## RESULTS

### Patient characteristics

**Figure 1:**
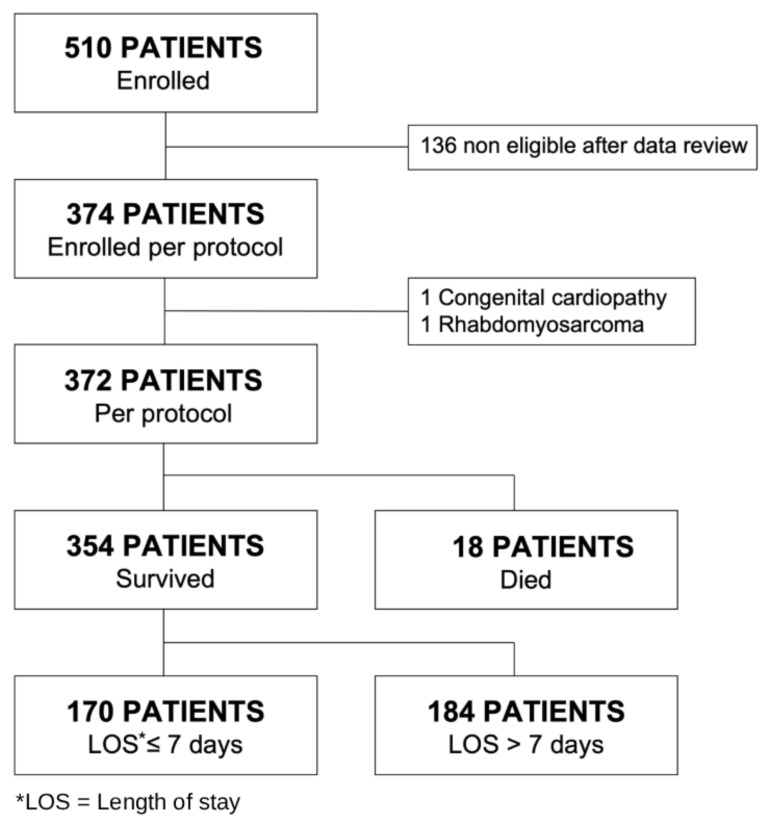
Patient flowchart.

During the study period, we initially evaluated 510 patients, of whom 372 met the inclusion criteria and were enrolled in the study (**Figure 1**). The MD age was 3.15 years (interquartile range = 1.55–6.21), and 48% (n/N = 177/372) were female (**Table 1**). Of all patients, 26% (n/N = 96/370) had sickle cell disease, 2% (n/N = 7/371) had HIV, and 1% (n/N = 3/372) had asthma. The overall mortality rate was 4.8% (n/N = 18/372) and MD duration of hospitalisation was 7.0 days (interquartile range = 3.0–14.0). Among those who survived, 52% (n/N = 184/354) had a hospitalisation duration >7 days. Only three patients were admitted to the intensive care unit, and two of them died.

**Table 1:** Baseline characteristics of the children hospitalised with pneumonia in Luanda

		LOS			Outcome		
	**All patients**	**≤7 days**	**>7 days**	***P*-value**	**Survived**	**Died**	***P*-value**
**Demographic characteristics, n (%)**							
Total number	372	170	184		354	18	
Age <1 year	51 (14)	29 (17)	16 (9)	0.018	45 (13)	6 (33)	0.025
Female	177 (48)	78 (46)	89 (48)	0.64	167 (47)	10 (56)	0.49
Black race	357/361 (99)	166/167 (99)	173/176 (98)	0.18	339/343 (99)	18/18 (100)	1.00
**Socioeconomic characteristics, n/N (%)**							
Breastfed (<2 years)	90/128 (70)	49/66 (74)	33/52 (63)	0.21	82/118 (69)	8/10 (80)	0.72
WAZ<−2SD	92/335 (27)	39/157 (25)	47/163 (29)	0.42	86/320 (27)	6/15 (40)	0.37
People in household >5	211/371 (57)	100/169 (59)	99/184 (54)	0.31	199/353 (56)	12/18 (67)	0.39
Smoking exposure*	82/308 (27)	33/134 (25)	47/158 (30)	0.33	80/292 (27)	2/16 (13)	0.25
Running water at home	167/370 (45)	81/168 (48)	81/184 (44)	0.43	162/352 (46)	5/18 (28)	0.13
Electricity at home	320/367 (87)	145/165 (88)	160/184 (87)	0.80	305/349 (87)	15/18 (83)	0.71
Cooking at home using gas or electric stove†	262/370 (71)	132/169 (78)	121/183 (66)	0.013	253/352 (72)	9/18 (50)	0.046
**Clinical characteristics, n/N (%)**							
SCD	96/370 (26)	53/169 (31)	42/183 (23)	0.076	95/352 (27)	1/18 (1)	0.051
HIV	7/371 (2)	3/170 (2)	4/183 (2)	1.00	7/353 (2)	0/18 (0)	1.00
Moderate or severe malnutrition‡	106/367 (29)	35/165 (21)	63/184 (34)	0.007	98/349 (28)	8/18 (44)	0.18
Previous pneumonia	95/347 (27)	42/161 (26)	47/169 (28)	0.72	89/330 (27)	6/17 (35)	0.42
Vaccinations reported as ‘up to date’	221/338 (65)	114/159 (72)	100/164 (61)	0.042	214/323 (66)	7/15 (47)	0.12
Previous doctor's appointments	252/361 (70)	101/163 (62)	135/180 (75)	0.009	236/343 (69)	16/18 (89)	0.070
Duration of illness before admission (in days), MD (IQR)	4.0 (2.0–9.0)	3.0 (2.0–6.0)	6.0 (3.0–12.0)	<0.001	4.0 (2.0–9.0)	4.0 (2.0–9.0)	0.67
**Reported symptoms before admission, n (%)**							
Cough	348 (94)	162 (95)	173 (94)	0.60	335 (95)	13 (72)	0.004
Stuffy nose	114 (31)	64 (38)	47 (26)	0.014	111 (31)	3 (17)	0.19
Chest pain	154 (41)	60 (35)	86 (47)	0.029	146 (41)	8 (44)	0.79
Diarrhoea	296 (80)	31 (18)	40 (22)	0.41	71 (20)	5 (28)	0.38
Vomiting	79 (21)	31 (18)	42 (23)	0.29	73 (21)	6 (33)	0.23
Lethargy	26 (7)	10 (6)	14 (8)	0.52	24 (7)	2 (11)	0.36
**Clinical signs on admission**							
Respiration on admission, n (%)							
*Normal*	44 (12)	22 (13)	21 (11)	0.66	43 (12)	1 (1)	0.71
*Inter- or subcostal retractions*	199 (53)	99 (58)	91 (49)	0.098	190 (54)	9 (50)	0.76
*Suprasternal retraction*	39 (10)	21 (12)	16 (9)	0.26	37 (10)	2 (11)	1.00
*Nasal flaring*	184 (49)	82 (48)	95 (52)	0.52	177 (50)	7 (39)	0.36
*Grunting*	79 (21)	28 (16)	45 (24)	0.064	73 (21)	6 (33)	0.23
Chest auscultation findings, n (%)							
*Normal*	7 (2)	3 (2)	4 (2)	1.00	7 (2)	0 (0)	1.00
*Rales*	116 (31)	66 (39)	47 (26)	0.007	113 (32)	3 (17)	0.17
*Crepitations*	166 (45)	74 (44)	84 (46)	0.69	158 (45)	8 (44)	0.99
*Wheezing*	39 (10)	22 (13)	15 (8)	0.14	37 (10)	2 (11)	1.00
*Diminished breath sounds*	193 (52)	60 (35)	125 (68)	<0.001	185 (52)	8 (44)	0.52
Other clinical signs, n/N (%)							
Seizures	16/358 (4)	4/165 (2)	9/176 (5)	0.20	13/341 (4)	3/17 (18)	0.034
Altered consciousness	21/369 (6)	7/169 (4)	10/182 (5)	0.56	17/351 (5)	4/18 (22)	0.014
Inability to drink	75/366 (20)	22/167 (13)	45/182 (25)	0.006	67/349 (19)	8/17 (47)	0.011
Oedema	39/364 (11)	10/164 (6)	26/182 (14)	0.013	36/346 (10)	3/18 (17)	0.42
O_2_ saturation <90%	38/352 (11)	18/158 (11)	18/176 (10)	0.73	36/334 (11)	2/18 (11)	1.00
Elevated respiratory rate for age on admission§	285/367 (78)	134/168 (80)	136/181 (75)	0.30	270/349 (77)	15 (83)	0.77
Temperature ≥38°C	134/369 (36)	60/170 (35)	70/181 (39)	0.51	130/351 (37)	4/18 (22)	0.20
**Radiological results, n/N (%)**							
Consolidation	255/363 (70)	94/167 (56)	148/181 (82)	<0.001	242/348 (70)	13/15 (87)	0.25
Atelectasis	258/362 (71)	92/166 (55)	152/181 (84)	<0.001	244/347 (70)	14/15 (93)	0.077
Diffuse infiltration	206/363 (57)	109/167 (65)	88/181 (49)	0.002	197/348 (57)	9/15 (60)	0.80
Pleural effusion	149/359 (42)	33/164 (20)	109/181 (60)	<0.001	142/345 (41)	7/14 (50)	0.51
**Laboratory results, MD (IQR)**							
Haemoglobin (g/dL)	7.3 (5.5–9.3)	7.2 (5.4-9.5)	7.4 (5.8–9.1)	0.49	7.3 (5.6–9.2)	8.3 (6.4–10.0)	0.12
WBC (1000/μL)	20.8 (11.0–25.6)	18.4 (10.8–24.0)	21.0 (11.5–25.6)	0.47	20.3 (11.2–25.5)	30.6 (10.2–32.7)	0.77
CRP at enrolment (mg/L)	131 (75-180)	112 (42–163)	142 (95–190)	<0.001	127 (71–178)	198 (168–250)	<0.001

CRP – C-reactive protein, HIV – human immunodeficiency virus, LOS – length of stay, SCD – sickle cell disease, WAZ – weight for age Z-score, WBC – white blood cell count

*Smoking exposure was defined as a caregiver reporting smoking indoors or outdoors at home or relatives smoking around the child.

†Compared to cooking at home using coal or wood.

‡Compared to no malnutrition.

§Pediatric Early Warning Score of ≥1.

At admission, 5% (n/N = 19/369) of patients had altered consciousness, and 1% (n/N = 2/369) were in a coma. Moderate malnutrition was observed in 20% (n/N = 74/367) of patients, while 9% (n/N = 32/367) had severe malnutrition. Data on duration of illness before hospitalisation were available for 97% (n/N = 360/372), CRP concentrations at enrolment for 95% (n/N = 355/372), white blood cell counts for 54% (n/N = 202/372), and blood haemoglobin for 69% (n/N = 258/372) of enrolled patients.

### Primary outcome: risk factors for mortality

We used two multivariable logistic regression models to examine mortality (**Table 2**). Model 1 (Nagelkerke R^2^ = 0.2575; accuracy = 0.9628; precision = 1.0000; AUC = 0.8155; Brier score = 0.0361) included three predictors, while model 2 included additional predictors added sequantially based on statistical significance, which improved the overall model performance (Nagelkerke R^2^ = 0.4395; accuracy = 0.9672; precision = 0.9969; AUC = 0.9123; Brier score = 0.0302). Based on these performance improvements, we selected Model 2 as the final multivariable model.

**Table 2:** Logistic regression results for mortality among children hospitalised with pneumonia

	Unadjusted model		Model 1* (n = 349)		Model 2* (n = 335)	
	**OR (95% CI)**	***P*-value**	**aOR (95% CI)**	***P*-value**	**aOR (95% CI)**	***P*-value**
**Age <1 year**	3.43 (1.15–9.32)	0.019			7.51 (1.72–34.12)	0.007
**Cough before hospitalisation**	0.15 (0.05–0.50)	<0.001	0.10 (0.03–0.40)	<0.001	0.05 (0.01–0.26)	<0.001
**Altered consciousness on admission**	5.62 (1.47–17.71)	0.0053			3.07 (0.36–20.39)	0.27
**Seizures on admission**	5.41 (1.15–19.26)	0.015			10.62 (1.17–80.71)	0.024
**Inability to drink on admission**	3.74 (1.36–10.14)	0.009	3.92 (1.30–12.04)	0.015	3.35 (0.88–12.89)	0.072
**CRP≥150 mg/L at enrollment**	6.21 (1.98–27.31)	0.005	9.71 (2.52–64.86)	0.004	27.77 (4.48–575.26)	0.004

aOR –adjusted odds ratio, CI – confidence interval, CRP – C-reactive protein, OR – odds ratio

*Adjusted for the other covariates included in each model.

**Figure 2:**
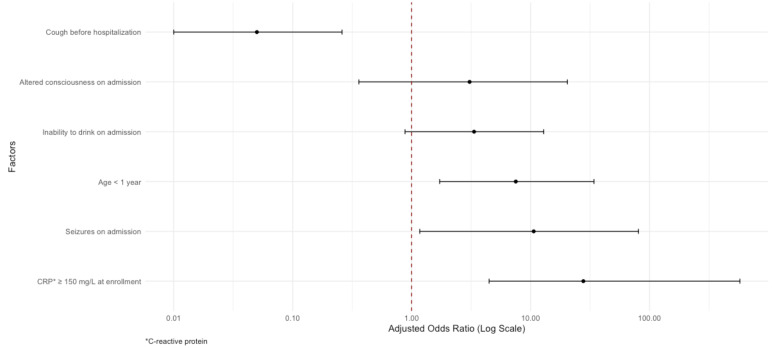
Logistic regression results for mortality among children hospitalised with pneumonia.

Six risk factors for in-hospital mortality were included in the final multivariable model (**Figure 2**). In the adjusted analysis, age <1 year (adjusted odds ratio (aOR) = 7.51; 95% confidence interval (CI) = 1.72–34.12, *P* = 0.007), seizures on admission (aOR = 10.62; 95% CI = 1.17–80.71, *P* = 0.024), and CRP*≥*150 mg/L at enrollment (aOR = 27.77; 95% CI = 4.48–575.26, *P* = 0.004) were associated with higher odds of mortality. Conversely, a history of cough before hospitalisation was associated with reduced odds of mortality (aOR = 0.05; 95% CI = 0.01–0.26, *P* < 0.001).

### Secondary outcome: risk factors for prolonged hospitalisation

We used two multivariable logistic regression models to examine prolonged hospitalisation (**Table 3**). Model 1 (Nagelkerke R^2^ = 0.3593; accuracy = 0.7407; precision = 0.7143; AUC = 0.8052; Brier score = 0.1792) included three predictors, while while model 2 included additional predictors added sequantially based on statistical significance, which improved the overall model performance (Nagelkerke R^2^ = 0.3914; accuracy = 0.7420; precision = 0.7027; AUC = 0.8224; Brier score = 0.1718). Further addition of predictors beyond model 2 did not result in meaningful improvement in model performance metrics. We also found no association between cooking method and malnutrition (χ^2^ = 0.1655; *P* = 0.6842), which justifies our choice of keeping them both in our final model. Based on this, we selected model 2 as the final multivariable model.

**Table 3:** Logistic regression results for hospitalisation duration >7 days among children hospitalised with pneumonia

	Unadjusted model		Model 1* (n = 324)		Model 2* (n = 314)	
	**OR (95% CI)**	***P*-value**	**aOR (95% CI)**	***P*-value**	**aOR (95% CI)**	***P*-value**
**Cooking at home using coal or wood†**	1.83 (1.14–2.96)	0.013			2.05 (1.13–3.80)	0.020
**Duration of symptoms before hospitalisation ≥5 days**	2.50 (1.62–3.87)	<0.001	1.90 (1.13–3.20)	0.015	1.73 (0.99–3.06)	0.056
**Previous doctor's appointments**	1.84 (1.16–2.94)	0.010			1.20 (0.64–2.25)	0.56
**Malnutrition on admission**	1.93 (1.20–3.15)	0.007	2.05 (1.14–3.74)	0.017	2.15 (1.17–4.01)	0.014
**Inability to drink on admission**	2.16 (1.25–3.85)	0.007	2.07 (1.07–4.10)	0.034	2.43 (1.22–5.00)	0.014
**Rales on chest auscultation on admission**	0.54 (0.34–0.85)	0.008			0.70 (0.39–1.25)	0.23
**Diminished breath sounds on chest auscultation on admission**	3.88 (2.51–6.07)	<0.001	2.45 (1.41–4.29)	0.001	2.67 (1.49–4.82)	0.001
**Consolidation or lobar infiltration on chest x-ray**	3.48 (2.16–5.71)	<0.001	1.08 (0.48–2.40)	0.86	0.96 (0.41–2.18)	0.92
**Atelectasis on chest x-ray**	4.22 (2.58–7.04)	<0.001	1.93 (0.87–4.34)	0.11	1.86 (0.82–4.24)	0.14
**Diffuse infiltration on chest x-ray**	0.50 (0.33–0.77)	0.002	0.88 (0.50–1.55)	0.65	0.89 (0.49–1.61)	0.70
**Pleural effusion on chest x-ray**	6.01 (3.74–9.86)	<0.001	3.84 (2.19–6.83)	<0.001	3.99 (2.23–7.27)	<0.001

aOR – adjusted odds ratio, CI – confidence interval, OR – odds ratio

*Adjusted for the other covariates included in each model.

†Compared to cooking at home using gas or electric stove.

**Figure 3:**
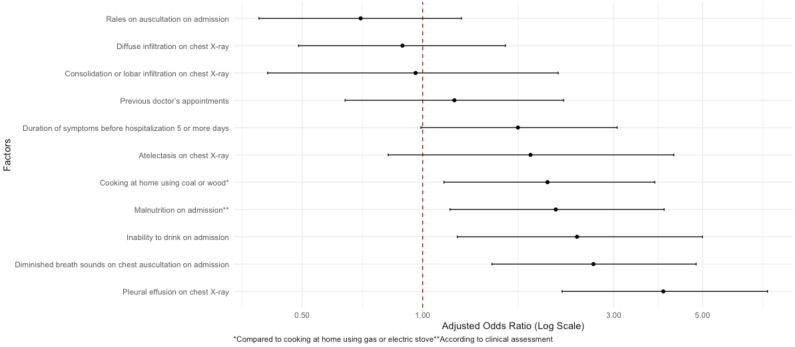
Logistic regression results for hospitalisation duration >7 days among children hospitalised with pneumonia.

A total of 11 risk factors for prolonged hospitalisation were included in the final multivariable model (**Figure 3**). In adjusted analysis, cooking at home with coal or wood (aOR = 2.05; 95% CI = 1.13–3.80, *P* = 0.020), malnutrition (aOR = 2.15; 95% CI = 1.17–4.01, *P* = 0.014), inability to drink on admission (aOR = 2.43; 95% CI = 1.22–5.00, *P* = 0.014), diminished breath sounds on auscultation (aOR = 2.67; 95% CI = 1.49–4.82, *P* = 0.001), and pleural effusion on chest x-ray (aOR = 3.99; 95% CI = 2.23–7.27, *P* < 0.001) were statistically significantly associated with increased odds for prolonged hospitalisation.

## DISCUSSION

To our knowledge, we are the first to explore the risk factors of severe CAP in children with pneumonia in Angola. To comprehensively assess disease severity, we selected mortality as the primary outcome and prolonged hospitalisation >7 days as the secondary outcome. Of all patients, 4.8% died, and the MD LOS was 7.0 days. These results are somewhat higher than those reported in other studies [13,14], possibly due to delays in treatment and high rates of associated pathologies.

The strongest risk factors for childhood pneumonia mortality in LMICs identified in previous studies include young age, inadequate immunisation, hypoxemia, altered consciousness, malnutrition, anaemia, and comorbidities [8]. In our cohort, factors significantly associated with mortality were young age, seizures, and elevated CRP at presentation, while cough before hospitalisation was protective. There is no widespread agreement on the main predictors of prolonged hospitalisation in children with pneumonia, and the literature is particularly limited in LMICs. We found cooking with coal or wood, malnutrition, inability to drink, diminished breath sounds, and the presence of pleural effusion to be significantly associated with prolonged hospitalisation.

Prior research, including a systematic review of multiple studies in LMICs [8], has established age <12 months as a significant risk factor for childhood pneumonia mortality (MD OR = 2.62; 95% CI = 1.37–28.5). Consistent with those findings, we found an association between age <1 year and increased odds of mortality. The vulnerability of this age group may be due to their still-developing immune system [15], smaller airways [16], and lack of muscle strength [17].

A limited number of studies have examined the association between seizures and mortality in paediatric CAP [18–20]. Araya *et al.* (OR = 10.6; 95% CI = 4.9–23) [18] and Macpherson *et al.* [20] (OR = 2.48; 95% CI = 1.65–3.72) found significant associations between seizures and morality; however, neither included seizures in multivariate models due to collinearity or a failure to improve the discriminatory power of the final model. Similarly, in a study by Ramachandran *et al.*, seizures increased the risk of mortality in univariate models (OR = 2.31; 95% CI = 0.84–6.35), but this effect was not sustained upon multivariable adjustment [19]. We found a significant association between seizures and mortality, which persisted in the multivariable analysis. Seizures in children with CAP may be attributed to hypoxia, metabolic derangements, sepsis, or cerebral inflammation [5].

CRP, an acute-phase protein synthesised in response to interleukin-6 [7], has been investigated as a potential predictor for childhood pneumonia severity, but results have varied. A systematic review by Wilkes *et al.* [8] reported that 33% (n/N = 2/6) of studies conducted in LMICs demonstrated a significant association between elevated CRP and childhood pneumonia mortality. More specifically, Diez-Padrisa *et al.* found a significant difference in CRP concentrations between survivors (MD CRP = 61 mg/L) and fatalities (MD CRP = 161 mg/L) [21]. However, an important overlap of values was observed between the two groups [21]. Shan *et al.* also reported a significant association between abnormal CRP concentrations (>8 mg/L) and mortality (aOR = 2.55; 95% CI = 1.80–3.61) [22]. Differing from the findings of several earlier studies, we found a significant association between CRP concentrations of ≥150 mg/L and increased odds of mortality, suggesting its potential relevance as a predictor of childhood pneumonia severity in this setting.

The relationship between cough and paediatric CAP mortality has not been thoroughly investigated. Shan *et al.* [22] found that cough was protective against intensive care unit admission (aOR = 0.28; 95% CI = 0.21–0.36), while Gallagher *et al.* found it to be protective against mortality (aOR = 0.48; 95% CI = 0.29–0.78) [23]. Consistently, we found cough before hospitalisation to be associated with reduced odds of mortality. This protective effect of cough suggests that its absence may indicate greater respiratory distress, or the presence of sepsis or malaria coinfection, which are associated with higher mortality rates than pneumonia in isolation [23].

Household air pollution resulting from the incomplete combustion of solid fuels (*e.g.* wood, dung, crop residues, and charcoal) has been strongly associated with an increased risk of acute lower respiratory infections in LMICs [24]. However, the association between the severity of childhood pneumonia and household air pollution has been scarcely investigated. In our study, cooking with coal or wood increased the odds of prolonged LOS compared to cooking with gas or an electric stove. This may be because coal or wood indoor air pollution can cause damage to the epithelial cell lining of the respiratory tract and/or suppress the host immune responses, facilitating the infection process caused by pneumonia pathogens [25].

The relationship between malnutrition and paediatric CAP mortality has been widely investigated, and there is a prevailing consensus that malnutrition constitutes a significant risk factor for mortality. Wilkes *et al.*' systematic review found malnutrition to be an independent risk factor in 80% (n/N = 16/20) of studies (MD OR = 3.71; 95% CI = 1.71–4.63) [8]. Although malnutrition and other outcome measures of CAP have not been as extensively studied, several studies have also demonstrated a significant association between malnutrition and prolonged hospitalisation [14,26,27]. Consistently, we found moderate or severe malnutrition to be significantly associated with prolonged hospitalisation. This is likely due to the high prevalence of macronutrient and micronutrient deficiencies in malnourished children, which weaken the immune system by impairing cell-mediated immunity and reducing secretory IgA production [28].

Gallagher *et al.* identified the inability to drink as a significant risk factor for childhood pneumonia mortality [23]. In a broader clinical context, inability to drink is recognised as a general danger sign associated with increased risk of death in children with severe infections [29]. Nonetheless, evidence regarding its association with duration of hospitalisation remains limited. We found that the inability to drink was associated with prolonged hospitalisation, likely due to the need for intravenous fluids or nasogastric tube until adequate oral intake could be re-established.

Diminished breath sounds are a common clinical finding in childhood pneumonia, yet their association with LOS has been infrequently studied. However, a recent investigation in high-income settings identified an association between decreased breath sounds and moderate or severe childhood CAP [30]. We found that diminished breath sounds were associated with prolonged hospitalisation. Reduced breath sounds can indicate pleural effusion or other complications of pneumonia [31], which may necessitate drainage, and hypoxic pneumonia [32], which often requires extended oxygen therapy, both of which can contribute to prolonged hospitalisation.

Multiple studies conducted in LMICs have demonstrated a significant association between pleural effusion and increased risk of mortality in paediatric CAP (OR = 2.6–6.96) [18,33,34], although its effect on LOS has been less extensively studied. However, in a multicentre cohort study conducted in the USA, the presence of pleural effusion on chest x-ray was significantly associated with a longer duration of hospitalisation (aOR = 2.6; 95% CI = 1.9–3.6) [35]. In agreement with those findings, pleural effusion on chest x-ray was significantly associated with increased odds of prolonged LOS in our study.

While our study provides valuable insights into the risk factors for prolonged hospitalisation and mortality in children hospitalised with CAP in Angola, several limitations must be acknowledged. First, we did not have complete data for all patients, and although missing data were assumed to be random, this may have introduced bias. Nonetheless, we conducted adjusted analyses and found several covariates associated with the outcomes. Second, the small number of deaths and the relatively small sample size limit strong conclusions. This was an exploratory study, and results should be interpreted as such, with future research needed to confirm the findings. Third, we excluded children with comorbidities aside from asthma, sickle cell disease, and HIV. Excluding those children may affect the generalisability of our findings to populations with a broader range of comorbid conditions. However, the excluded comorbidities are rare in children, and we believe our cohort sufficiently represents the general population. Additionally, the lack of standardised discharge criteria may have influenced hospitalisation duration. However, this likely had minimal impact, as the same clinical team delivered care throughout the study period. Duration of hospitalisation should be seen as a composite outcome rather than a direct measure of physiological severity. Furthemore, potential misclassification and measurement errors cannot be fully excluded. However, data were collected using standardised clinical procedures, so any bias is likely minimal and non-differential. Moreover, despite the overlap with the COVID-19 pandemic, enrollment volumes remained stable throughout the study period, and we did not observe major differences in in-hospital mortality or LOS, although residual confounding related to pandemic-era healthcare changes cannot be fully excluded. Lastly, our cohort only included hospitalised patients in a single centre in Angola. Future studies including ambulatory and hospitalised children from diverse geographic locations are warranted.

## CONCLUSIONS

We identified young age, indoor cooking with coal/wood, inability to drink, malnutrition, seizures, diminished breath sounds, pleural effusion, and high CRP as key risk factors for severe CAP in Angolan children. Our findings emphasise the need to prevent malnutrition and reduce indoor air pollution, although the association with household fuel type should be interpreted cautiously due to possible residual socioeconomic confounding. Additionally, laboratory and clinical parameters may serve as valuable indicators of pneumonia severity in resource-limited settings to guide treatment decisions. In this context, CRP is routinely available as a qualitative test (positive/negative), and quantitative CRP measurements could help better identify children at the highest risk of severe disease, although optimal clinical thresholds would still require validation. These results should be interpreted in the context of a single tertiary hospital in Luanda, where health system factors may influence outcomes.

## Additional material


Online Supplementary Document
